# Genetic Variations in *IL28B* and Allergic Disease in Children

**DOI:** 10.1371/journal.pone.0030607

**Published:** 2012-01-25

**Authors:** Silvana Gaudieri, Michaela Lucas, Andrew Lucas, Elizabeth McKinnon, Hiba Albloushi, Andri Rauch, Julia di Iulio, David Martino, Susan L. Prescott, Meri K. Tulic

**Affiliations:** 1 School of Anatomy and Human Biology, University of Western Australia, Nedlands, Australia; 2 Centre for Forensic Science, University of Western Australia, Nedlands, Australia; 3 Institute for Immunology and Infectious Diseases, Murdoch University, Murdoch, Australia; 4 PathWest and Laboratory Medicine, University of Western Australia, Nedlands, Australia; 5 University Clinic of Infectious Diseases, University Hospital Bern and University of Bern, Bern, Switzerland; 6 Institute of Microbiology, University Hospital Centre and University of Lausanne, Lausanne, Switzerland; 7 School of Paediatrics and Child Health, University of Western Australia, Nedlands, Australia; Rush University, United States of America

## Abstract

Environmental changes affecting the relationship between the developing immune system and microbial exposure have been implicated in the epidemic rise of allergic disease in developed countries. While early developmental differences in T cell function are well-recognised, there is now emerging evidence that this is related to developmental differences in innate immune function. In this study we sought to examine if differences associated with innate immunity contribute to the altered immune programming recognised in allergic children. Here, we describe for the first time, the association of carriage of the T allele of the tagging single nucleotide polymorphism rs12979860 3 kb upstream of *IL28B*, encoding the potent innate immune modulator type III interferon lambda (IFN-λ3), and allergy in children (p = 0.004; OR 4.56). Strikingly, the association between rs12979860 genotype and allergic disease is enhanced in girls. Furthermore, carriage of the T allele at rs12979860 correlates with differences in the pro-inflammatory profile during the first five years of life suggesting this contributes to the key differences in subsequent innate immune development in children who develop allergic disease. In the context of rising rates of disease, these immunologic differences already present at birth imply very early interaction between genetic predisposition and prenatal environmental influences.

## Introduction

The threat of premature death caused by infectious diseases in developed countries has largely past since the widespread introduction of antibiotics and improved standards of personal cleanliness, yet paradoxically contemporary communities are now beset by a dramatic increase in the burden of chronic inflammatory diseases. This clearly highlights the susceptibility of the immune system to modern environmental changes [1]. Not only allergic conditions such as asthma, allergic rhinoconjunctivitis, food allergies and eczema are on the rise but autoimmune disorders (such as type 1 diabetes and chronic inflammatory bowel disease) as well as neurodegenerative and cardiovascular diseases [1], are now following the same alarming trends [2]. While complex lifestyle changes are driving this surge in disease, genetic factors are critically important in determining the susceptibility to environmental changes that can enhance inflammatory responses, and the predisposition to these diverse inflammatory diseases [1]. Furthermore, the rising rates of allergic disease in infancy clearly indicates that early gene-environment interactions, even before birth, play an important role in determining susceptibility to inflammation and immune disease [3].

Prospective longitudinal case-control comparisons of immune development in well-characterized children provide opportunities to assess development of innate inflammatory responses in relationship to genetic and clinical phenotype. Using this approach, our initial studies [4], [5] revealed striking differences in the biological responses (to microbial products) of children who develop allergic disease. These children show increased inflammatory (IL-6, IL-1β and TNFα) cytokine production at birth and during their neonatal period, compared to children that remain non-allergic [4], [5] and this is associated with subsequent propensity for allergic Type 2 adaptive responses [5]. Plasmacytoid dendritic cells (pDC) from these allergic children express high levels of bacterial recognition receptor, toll like receptor (TLR)2, during their first year of life compared to non-allergic controls [5]. In the children with allergy high expression of TLR2^+^ on pDCs was strongly associated with a distinct innate immune response and allergic disease phenotype.

This new evidence that susceptibility to allergy may be associated with an altered innate immune development from birth prompts more detailed examination of underlying candidate pathways. Given clues that pDC function, which is pivotal in translating distinct innate stimuli into robust type I and III interferon (IFN) production, is affected in allergic children, we hypothesised that an alteration in the biology of type I and/or type III IFN might also be affected.

The newly discovered type III IFN family is composed of three closely related molecules in humans: IFN-λ1 (IL29), IFN-λ2 (IL28A), and IFN-λ3 (IL28B) that exhibit anti-viral activity [6] *via* a similar but likely independent mechanism to type I IFNs. These molecules are likely to have an immunomodulatory function as studies have shown that IFN-λ1-treated human DCs can induce proliferation of suppressor T cells [7], inhibit *in vitro* naïve and memory IL-13 responses in human T cells [8], [9], augment antigen-specific IFNγ release [10], and upregulate TLR expression and their response to ligands [11]. Support for the role of IFN-λ in allergic disease can be extrapolated from relatively few studies that have examined their role in allergic asthma. Contoli and colleagues have shown that deficient IFN-λ induction (IFN-λ1 and IFN-λ2/3) by viral (rhinovirus) and bacterial (lipopolysaccharide) stimuli was associated with severity of asthma exacerbations in allergic asthmatics [12]. A recent study clearly demonstrated that IFN-λ2 can effectively induce type 1 immunity and suppresses Th2 immunity and consequently allergic airway disease in a mouse model of allergic asthma *via* its action on DCs [13].

Furthermore, genetic variations in *IL28B* (IFN-λ3) have been linked with differential expression of interferon-stimulated genes (ISG) in patients with chronic Hepatitis C [14], [15] and with their outcome of IFN-α therapy [16], [17]. Although the IFN-λ family comprises of the three related members, IFN-λ3 is to date the only member with genetic variations associated with differential expression profiles for downstream genes involved in the immune response and outcome for a disease that exhibits symptoms of dysregulation of the immune response. Accordingly, in this study we set to examine the relationship between *IL28B* genetic variation and allergic disease in children.

## Results

### Genetic variation at the tagging SNP rs12979680 3 kb upstream of *IL28B* is associated with risk of allergic disease

**Figure 1:**
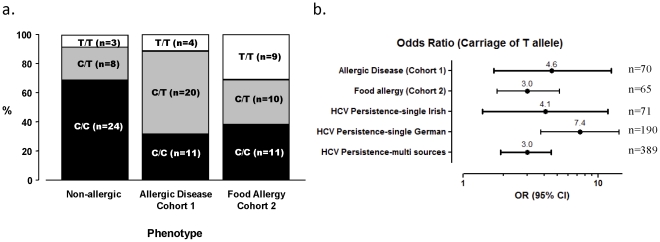
Variation at tagging SNP rs12979860 confers risk for allergic disease. **a.** Carriage of the T allele of rs12979860 is over-represented in children with allergic disease (cohort 1, n = 70) (p = 0.004). This relationship is also observed for children with IgE-mediated food allergy (Cohort 2, n = 60) (p = 0.04 dominant model). For both cohorts, the frequency of the different genotypes varies between disease and control subjects (p = 0.02; cohort 1 and cohort 2). **b.** Odds ratio (OR; 95% CI) for carriage of T allele of rs12979860 and allergic disease and for HCV persistence. Odds ratio values for HCV cohorts are from di Iulio et al [20] and Tillman et al [28]. ORs for allergic disease and food allergy adjusted for gender. ORs for HCV cohorts adjusted for HBV co-infection in all cohorts and gender in the multiple source cohort.

We present data showing support for *IL28B* playing a role in allergic disease. We genotyped 70 children (35 allergic and 35 non-allergic; Cohort 1) for the tagging SNP rs12979860 that resides 3 kb upstream of *IL28B*. Carriage of the T allele at rs12979860 has previously been shown to be highly predictive of poor Hepatitis C virus (HCV) infection and treatment outcome [16]–[19]. Here, presence of the T allele at rs12979860 was over-represented in children with allergic disease (p = 0.004; OR = 4.56, CI 1.7–12.6) (Fig. 1a). Although the frequency of the C allele was lower in allergic children compared to non-allergic children (0.6 vs 0.8, respectively) the strongest predictor of allergic disease was carriage of the T allele. The odds ratio obtained for this relationship (OR = 4.6) is akin to what was observed in a recent study examining the relationship between *IL28B* and HCV infection outcome in a single source cohort of similar size (OR = 4.1) (Fig. 1b). The single source cohort comprises women who were exposed to HCV contaminated immunoglobulin D in Ireland in the 1970s and inherently controls for ascertainment bias, clinical, demographic and viral factors that allow the optimal platform to assess the *IL28B* genetic contribution to HCV outcome. In studies that cannot account for these factors, the odds ratios obtained for the association of *IL28B* variants and HCV outcome are much lower (OR = 3.0 vs OR 4.1 and 7.4) [20]. In this study, the strict criteria used to assess allergic and non-allergic (non-atopic) children removed several confounding factors that allowed a better assessment of host genetic contribution to allergic disease. As sensitisation against environmental allergens in the Australian population is highly prevalent, the criteria used to select non-allergic children is critical and in this cohort selection for non-atopic children was based on an extensive array of clinical tests over the first five years of life; a critical period of immune ontogeny. Conservative assessment of the phenotype definition can offset the value of larger sample size in disease gene association studies [21]. Accordingly, the robust relationship we find between *IL28B* and allergic disease in a relatively small sample size suggests that *IL28B* (and effects on IFNλ-3 levels) is likely to play a dominant role in the susceptibility and development of allergic disease. These results are comparable to what has been reported for HCV infection and treatment outcome in which it is well established that *IL28B* variants are the strongest host genetic contributor to HCV outcome.

### Association between rs12979860 genotype and food allergy: Validation of genetic association in food allergic cohort

We then extended our analysis between allergic phenotype and carriage of the T allele at rs12979860 to a second paediatric cohort (n = 30; Cohort 2) in which subjects were defined as clinically food allergic. These subjects were recruited from the same clinic and therefore have similar demographic characteristics as Cohort 1. When we compared these subjects to the screened non-allergic subjects (controls) as described above we found that carriage of the T allele at rs12979860 is also associated with food allergy (p = 0.04; OR = 3.0, CI 1.8–5.2) (Fig. 1b). Interestingly for Cohort 2 but not Cohort 1, the frequency of the homozygote T/T genotype was higher than observed in the non-allergic children (Fig. 1a). Accordingly, all subsequent analyses performed on Cohort 1 examined the influence of carriage of the T “risk” allele.

### Association of rs12979860 genotype with allergic disease increases with age

**Figure 2:**
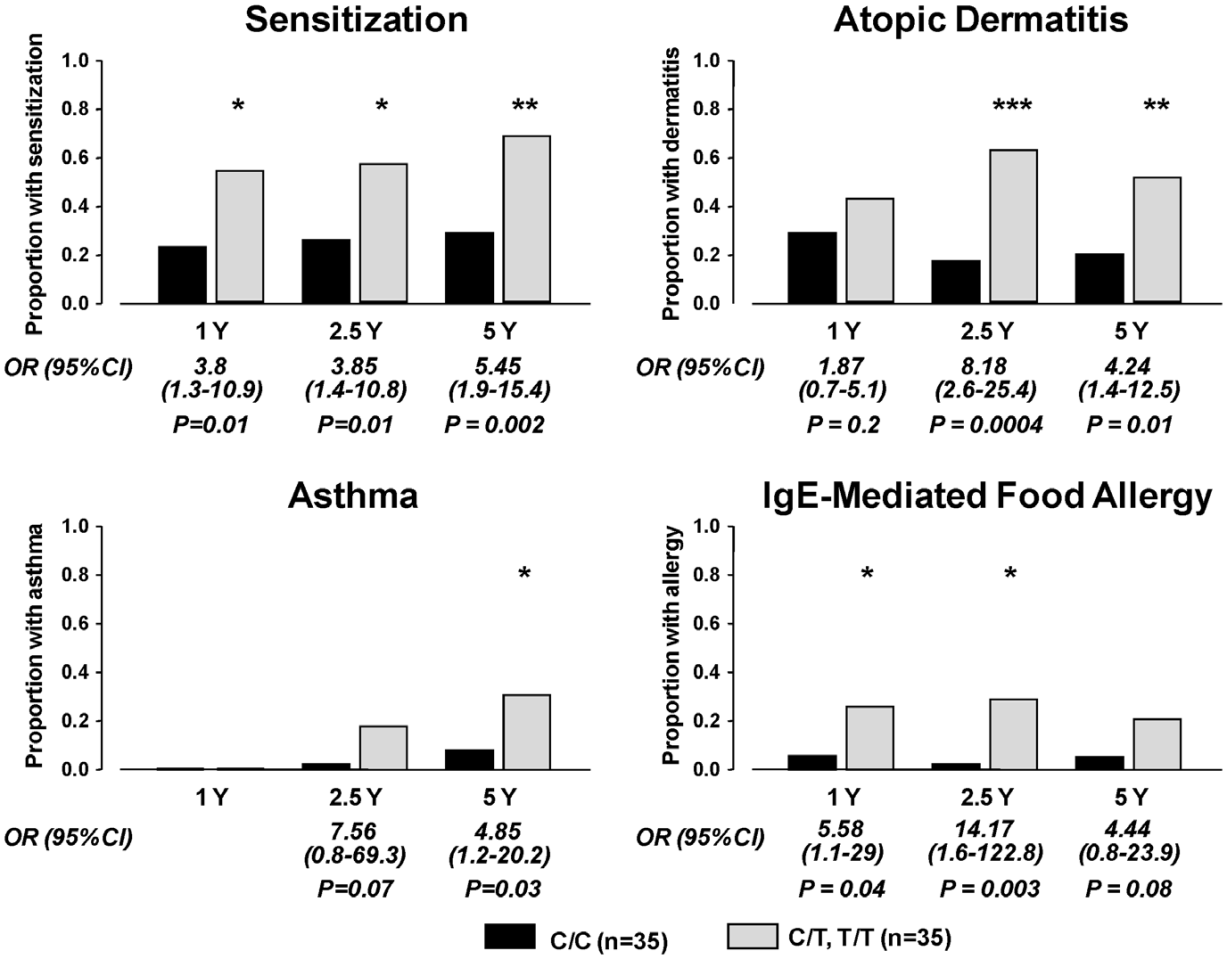
Carriage of the T allele of rs12979860 is associated with clinical presentation of allergic disease. Proportion of infants in cohort 1 that are sensitised, have doctor-diagnosed atopic dermatitis, asthma or IgE-mediated food allergy at 1, 2.5 or 5 years of age, with C/C (n = 35, black bars) or C/T, T/T (n = 35, grey bars) genotype. OR = odds-ratio. *P<0.05, **P<0.01 and ***P<0.001 versus age-matched C/C genotype.

In order to assess the impact of *IL28B* on early allergic phenotype, we examined the association of rs12979860 genotype with allergic outcome during early childhood using subjects in Cohort 1, as detailed clinical data on these subjects had been collected from follow-up visits during the first five years of life. There is a clear association between carriage of the T allele of rs12979860 and development of early allergic disease, particularly for sensitization against environmental antigens and IgE-mediated food allergy (Fig. 2). In the case of atopic dermatitis, there is a trend for discrimination between the rs12979860 genotype during the first year of life and this becomes significant from 2.5 years of age (Fig. 2). Clear diagnosis of asthma cannot be determined during the first year of life however carriage of the T allele is significantly associated with asthma diagnosis by age 5.

### Gender bias in the association of rs12979860 genotype with allergic disease

**Figure 3:**
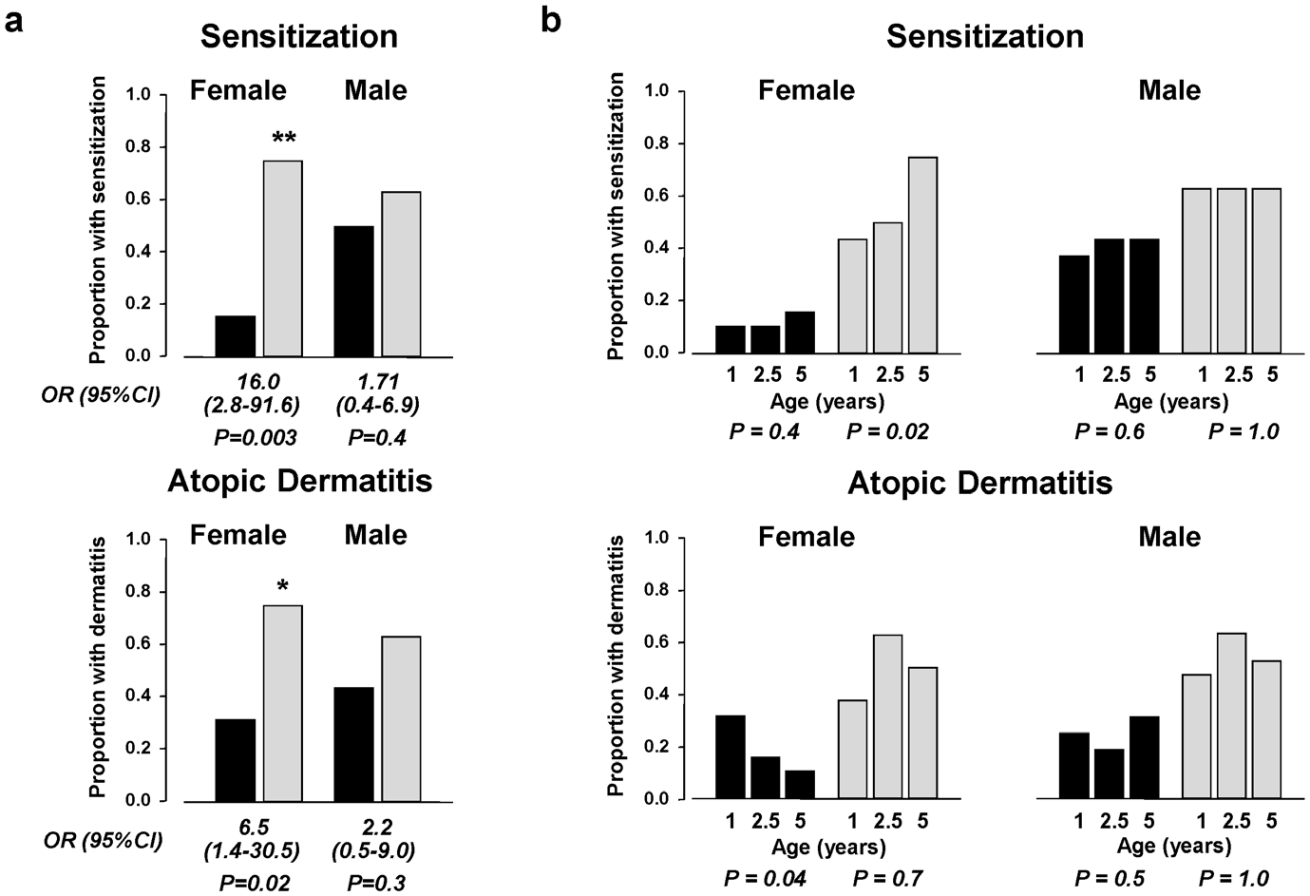
Gender differences in the association between carriage of the T allele of rs12979860 and allergic disease. **a.** Interaction between gender and carriage of the T allele of rs12979860 for sensitization (top panel) and atopic dermatitis (bottom panel) in cohort 1 in individuals with C/C (n = 35, black bars) or C/T,T/T (n = 35, grey bars) genotype. *P<0.05 and **P<0.001 versus females with C/C genotype. **b.** Trends in sensitization (top panel) and atopic dermatitis (bottom panel) levels with age relative to rs12979860 genotype. *P<0.05 versus proportion of sensitised females with C/C genotype and †P<0.05 versus C/T,T/T genotype in females with atopic dermatitis. OR = odds ratio.

There is a significant interaction between gender and carriage of the T allele of rs12979860 for allergic disease (P = 0.04). The effect is particularly evident for sensitization (Fig. 3a top) and to a lesser extent for atopic dermatitis (Fig. 3a bottom) but not for asthma or IgE-mediated food allergy during early childhood (data not shown). Interestingly, it was only amongst females that we found differential trends in allergy status with age relative to rs12979860 genotype. Carriage of the T allele of rs12979860 in females but not males was associated with increased risk of sensitisation with age (P = 0.02; Fig. 3b). Conversely, amongst females with the C/C genotype we see a reduction in the proportion with atopic dermatitis with age (P = 0.04; Fig. 3b).

### rs12979860 genotype correlates with innate immune function

**Figure 4:**
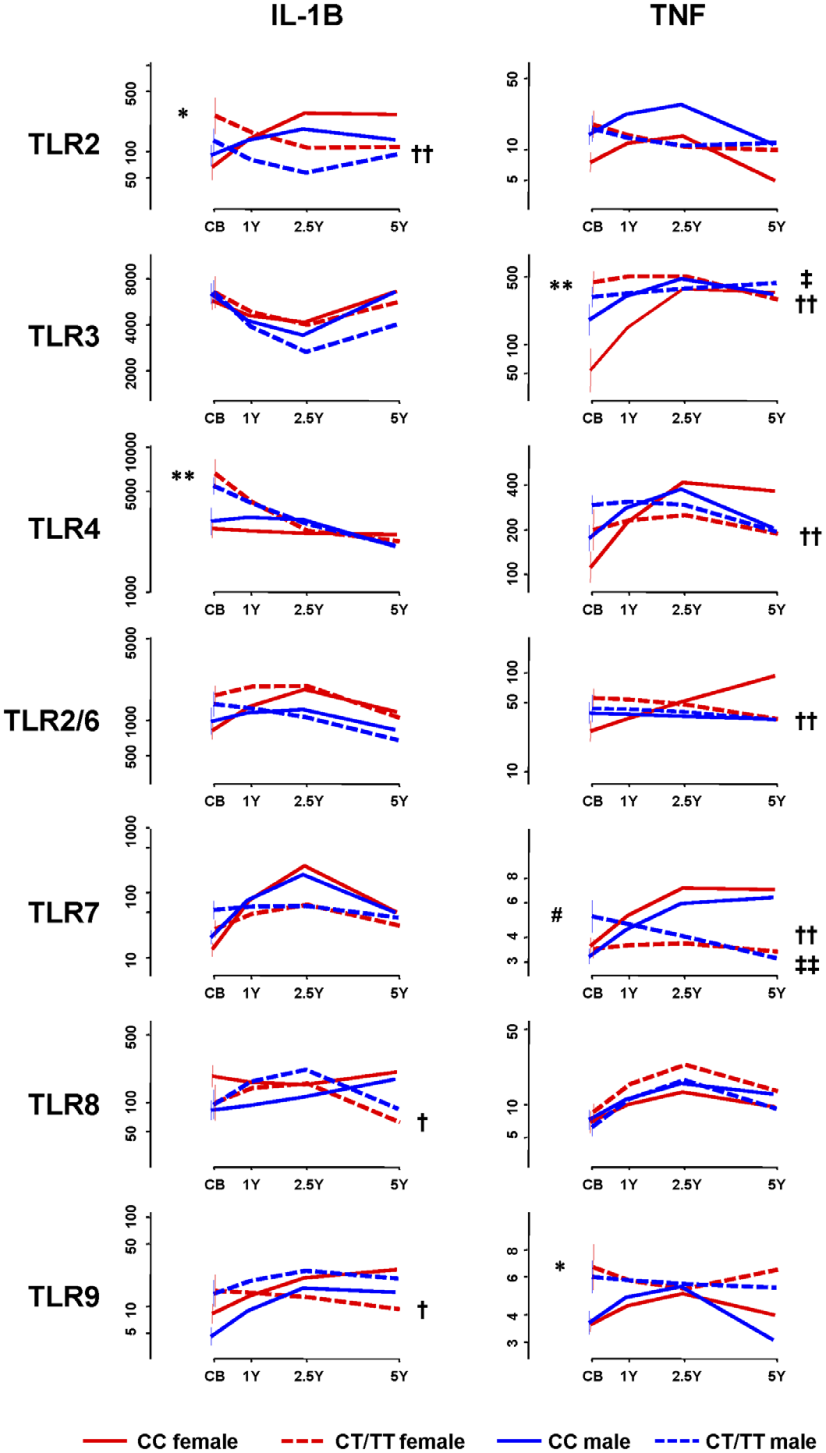
IL-1B and TNF cytokine production with age in peripheral blood mononuclear cells from individuals with C/C or C/T,T/T genotype stimulated with TLR agonists. Plots are scaled to span ∼90% of the data range. Differences between groups with p-values<0.05 are annotated: CB measures of rs12979860 genotype C/T, T/T vs C/C amongst females (*: P≤0.01) and males (#: P≤0.01); average linear trend of profiles of C/T,T/T vs C/C amongst females (†: P≤0.01, ††: P≤0.001) and males (‡: P≤0.01, ‡‡: P<0.001).

We then examined the association between TLR-mediated innate immune function (namely IL-1B, TNF alpha cytokine production after TLR ligation with classical model ligands [5]) and rs12979860 genotype. We have previously shown a differential in the level of pro-inflammatory cytokine production after cord blood stimulation with select TLR ligands is associated with either allergic and non-allergic status in later life [5]. Briefly, children with allergic disease have higher levels of pro-inflammatory cytokines at birth (in cord blood) and during their early postnatal period, however this responsiveness declines with age and by pre-school age (5 years) is significantly depressed compared to age-matched non-allergic controls. In non-allergic children, TLR responses mature with age, which is necessary to elicit appropriate responses to pathogens. Here we show that this key difference in immune ontogeny is significantly associated with rs12979860 genotype and can be seen after the stimulation with the majority of TLR ligands (Fig. 4). In general, subjects with the C/C genotype (solid lines) are characterised by significantly lower levels of pro-inflammatory cytokine production at birth after TLR-dependent stimulation (exception being TLR8). For these individuals with the C/C genotype, a postnatal increase in IL-1β and TNF-α production following TLR stimulation (most pronounced for TLR2, 3, 4 and 7) is observed (Fig. 4). These findings are contrasted by the profile of TLR-dependent cytokine responses in children carrying the T “risk” allele (that is, C/T or T/T genotype; dashed lines) (Fig. 4), which shows high levels of pro-inflammatory cytokines at birth (in cord blood) in response to TLR ligation. Furthermore, TLR-induced pro-inflammatory cytokine responses in these children decline with age (in particular TNF production). These findings are consistent with our previous observation in the same cohort showing a similar distinguishing pattern in cytokine profiles in allergic and non-allergic during the maturation of the immune system over the first five years of life [5]. Although this can be seen in male and female children, these trends are most pronounced in females with the C/C genotype (Fig. 4).

## Discussion

Allergic disease is a complex trait with a substantial genetic contribution likely involving a number of different genes, gene-gene interactions and environmental influences. However, identifying the causative genes involved in the susceptibility and progression of allergic disease is hampered by heterogeneity in clinical phenotype, incomplete penetrance, typical modest or low genetic effect size of a single causative gene and the often immeasurable interaction between genes and the environment. Importantly, phenotypic definition is a critical factor to consider in association studies to optimise the ability to identify new genetic associations with disease [21]. In this study, the use of stringent criteria to define allergic and non-allergic children during the critical five-year follow-up from birth has resulted in a clinically well-differentiated disease and control group that should facilitate the identification of putative causative genes in allergic disease.

Our previous studies have clearly shown a different developmental trajectory for innate immune function between allergic and non-allergic children during early childhood [4], [5]. Given the likely role of pDCs and IFNs in this framework, we investigated the role of genetic variations in *IL28B* in allergic disease. We show for the first time a strong association between genetic variations flanking and within *IL28B* and allergic disease. The effect of *IL28B* variations on atopy risk may be one of the strongest genetic predictors of allergic disease as most other candidate genes for allergic disease appear to have only a modest effect size. Interestingly, of more than a dozen genome-wide scans performed on atopic disease (including atopy, asthma and atopic dermatitis) none have highlighted this chromosomal region (www.genome.gov/gwastudies) as has been done for HCV infection and treatment outcome. This may be best explained by the clinical difficulty in phenotyping atopic and non-atopic subjects (particularly children) included in previous studies, given the high rate of atopy even in non-symptomatic subjects.

As an interesting analogy, HCV clearance (natural and treatment-induced) is associated with the C/C “protective” genotype of rs12979860 with a similar effect size. It currently remains speculative if the C/C genotype leads to higher or lower levels of IFN-λ (and specifically IFN-λ3) secretion (locally and systemically). Some studies have suggested that the C/C genotype is associated with diminished ISG expression (albeit mainly for liver and in HCV-infected subjects) [14], [15]. Others have shown higher levels of IFN-λ in plasma in individuals who carry the C allele of rs12979860 (C/C and C/T subjects) [22]. Interestingly, subjects with autosomal dominant STAT3 deficiency (Hyper IgE syndrome) [23], which may lead to mutations in the IFN-λ signalling pathway, present with atopic dermatitis and high levels of IgE in serum. Thus, one could speculate that the C/T or T/T genotype in allergic children is associated with lower levels of IFN-λ secretion. Furthermore, IFN-λ is thought to suppress Th2-type immune responses, which are the hallmark of allergic disease. This is further supported by the recent study by Koltsida and colleagues who showed that IFN-λ can promote Th1 immunity and suppress Th2 responses in the mouse model of allergic asthma [13] initiating suggestions that IFN-λ could be used as a new treatment approach for allergic asthma [24]. In this study, we have demonstrated a relationship between higher levels of a pro-inflammatory cytokine profile at birth with diminished levels of these cytokines over time in children who carry the T allele of rs12979860 with an inverse relationship observed for children with the “protective” C/C genotype. However, levels of IFN-λ3 at relevant sites for atopic disease have not been established and the role of IFN-λ3 in IgE-mediated diseases and in the Th1/Th2 balance remains of great interest.

Frequencies of the rs12979860 alleles vary across populations with Africans having a higher frequency of the T “risk” allele than Caucasians and Asians [19]. In HCV infection and treatment outcome, frequency of the T allele of rs12979860 correlates with the known disparity in responses in different populations [19]. In allergic disease, it is known that there are differences in the prevalence and disease progression of allergic disease in populations [2] with African Americans shown to have much higher rates of death from asthma. A better link between *IL28B* allele frequencies and epidemiological studies may reveal how much *IL28B* variation contributes to ethnic disparities in the prevalence of atopic disease.

Here, we also clearly show a gender bias in the “protective” effect of the C/C genotype of rs12979860 in females compared to males in atopic disease. The gender bias extends to the relationship between rs12979860 and innate immune function. The reasons for this gender bias are not clear but may involve differential transcriptional control or hormonal responsiveness of relevant genes. Interestingly, female gender is also a predictor of favourable HCV infection outcome [25].

In view of the increasing burden of allergic disease in clinical practice, novel biomarkers that can be used for risk stratification of challenges, treatment and diet advice are invaluable. Definition of the *IL28B* genotype in children will allow the rapid identification of those children that are at risk of developing allergic disease and therefore management/intervention can be optimised accordingly. Overall, analysing the *IL28B* genotype is a simple cost-effective test that can be automated and already has been optimised for clinical practice as a predictive factor for outcome of HCV infection.

In conclusion, despite a proposed linkage between the level of environmental pathogenic exposure and allergy (hygiene hypothesis) no clear mechanistic linkage has been proposed that might explain the apparent enhanced susceptibility. Here for the first time we show that genetic variants in an innate microbial defence pathway (*IL28B*) are highly predictive of allergic disease with clear gender bias, and directly correlated with the innate immune function. These findings have broad-ranging implications for understanding the pathogenesis of not only allergic disease but many other inflammatory and autoimmune diseases.

## Methods

### Subjects

All children were recruited ante-natally from healthy pregnant mothers from obstetricians in Perth, Australia. Children were selected from 739 children enrolled in prospective birth cohorts designed to examine allergy pathogenesis. The first cohort (Cohort 1) consisted of 35 allergic and 35 non-allergic infants, which were followed from birth to age 5 years to assess their development of innate immune function and are described elsewhere [5]. Children were defined as non-allergic if they had no history of sensitisation or allergic disease at any age (based on at least 2 follow-up visits), or allergic if they have had a doctor-diagnosis of atopic dermatitis, allergic rhinitis, food allergy or asthma, *and* specific IgE to allergens as detected by a skin-prick test (SPT) at one or more clinic visits. Cohort 2 consisted of children with doctor-diagnosed food allergy (n = 30) recruited from the same research clinic as Cohort 1. IgE-mediated food-allergy was defined as a history of immediate symptoms after contact with food, ingestion of food, or both *and* a positive SPT to that food. Symptoms of acute food allergy included skin reactions (hives, rash, or swelling) and/or respiratory tract symptoms (cough, wheeze, or stridor) and/or gastrointestinal symptoms (abdominal pain, vomiting, or loose stools) and/or cardiovascular symptoms (collapse). In this cohort, 12/30 (40%) had severe food allergy (resulting in anaphylaxis). 27/30 (90%) had allergy to eggs, 13/30 (43%) allergy to nuts, 7/30 (23%) were allergic to house dust mite, 5/30 (17%) to cat dander and 20/30 (67%) reacted to multiple foods. Subjects in Cohort 1 and 2 were all of Caucasian ethnicity.

### Ethics statement

Written, informed consent from the next of kin, carers or guardians on the behalf of the children participants was obtained for this study. Ethics approval was given by Princess Margaret Hospital Ethics Committee (EC07-74) and contributing Centres (EC2004/005). The protocol and the procedures of the study were conducted in conformity with the ethical guidelines of the Declaration of Helsinki.

### *IL28B* Genotyping

Genotyping of the tagSNP rs12979860 upstream of *IL28B* on subjects in both cohorts was performed by TaqMan allelic discrimination using the Custom Assay designed by Ge and colleagues [16]. Typing of rs12979860 was performed by two independent laboratories blinded to the clinical phenotype with 100% concordance. There was no significant deviation from Hardy Weinberg equilibrium.

Further characterisation of the *IL28B* region was performed by examining an additional set of putative causal SNPs (rs8403219, rs28416813, rs8103142 and rs4803217) that reside within the promoter, coding and 3′ untranslated region of *IL28B* [20]. These SNPs are in strong linkage disequilibrium with rs12979860 and define the two main haplotypes of the block encompassing *IL28B*, with the type II haplotype containing the minor or “risk” allele(s) for at least one of the putative causal SNPs. As there was complete linkage disequilibrium between rs12979680 and the type II haplotype in all subjects, the p-value and OR were the same as for carriage of the T allele of rs12979860. Genotyping of the additional four SNPs within *IL28B* and another tagging SNP rs8099917 10 kb upstream of *IL28B* on subjects in Cohort 1 and 2 was performed by TaqMan allelic discrimination using Custom Assays and Assay on demand provided by Applied Biosystems as described by di Iulio *et al* [20].

### Statistical analyses

Logistic regression was used to assess relative effects of genotype, gender and age on allergy status. Confidence intervals for odds ratios (OR) were obtained by exponentiating the 95% confidence interval for the log odds, estimated by the model coefficient, and p-values were determined by a Wald test. Multivariable models provided adjusted estimates and enabled assessment of variable interactions. Evaluation of trends in allergy status over age accommodated the within-individual correlations by assuming an autoregressive structure within a generalized estimating equation framework.

Cytokine measures were log-transformed prior to analysis to ensure approximate normality of residuals about mean values. Differences in baseline (cord blood) measures were assessed by standard linear regression. Mixed effect linear regression models were utilized for estimation of group-average changes in the measures over time since birth, and assessment of genotype and gender effects on the linear trend [26], [27]. The models assumed an autoregressive correlation structure for accommodation of the within-individual dependencies. All statistical analyses were carried out using TIBCO Spotfire S+ 8.2 for Windows (TIBCO Spotfire Inc, Palo Alto 2010).
